# Microbially derived biosensors for diagnosis, monitoring and epidemiology

**DOI:** 10.1111/1751-7915.12791

**Published:** 2017-08-03

**Authors:** Hung‐Ju Chang, Peter L. Voyvodic, Ana Zúñiga, Jérôme Bonnet

**Affiliations:** ^1^ Centre de Biochimie Structurale INSERM U1054 CNRS UMR5048 University of Montpellier Montpellier France

## Abstract

Living cells have evolved to detect and process various signals and can self‐replicate, presenting an attractive platform for engineering scalable and affordable biosensing devices. Microbes are perfect candidates: they are inexpensive and easy to manipulate and store. Recent advances in synthetic biology promise to streamline the engineering of microbial biosensors with unprecedented capabilities. Here we review the applications of microbially‐derived biosensors with a focus on environmental monitoring and healthcare applications. We also identify critical challenges that need to be addressed in order to translate the potential of synthetic microbial biosensors into large‐scale, real‐world applications.

Part of achieving the UN sustainable development goal #3 (‘Good health and well being’) relies on using biosensing technologies for the detection of environmental hazards or early diagnostics of diseases. As the majority of target populations live in developing countries, the next generation of biosensors need to solve the conundrum of being cost‐effective and easy to operate, while remaining highly sensitive and specific. Microbes could help address this challenge by providing a robust and inexpensive self‐manufacturing platform capable of integrating various physical and chemical signals. Here, we focus on how microbially‐derived biosensors can provide a solution to problems in environmental monitoring of harmful substances and in medical diagnostics. We present developments based on bacterial‐ as well as yeast‐based whole‐cell biosensors and conclude with recently developed cell‐free biosensors.

## Whole‐cell biosensors for environmental and food monitoring

The widespread use of chemicals in industry and agriculture has led to an increased environmental release of toxic compounds and subsequent food contamination. Consequently, sensitive, rapid, reliable and cost‐effective tools are needed to detect these toxic compounds and contribute to pollution mitigation programmes and treatment strategies. Traditionally, environmental pollutants have been measured by chromatographic methods (Liu *et al*., 2010). However, these technologies are expensive and require specialized equipments and well‐trained users. In addition, important parameters such as bioavailability, toxicity and genotoxicity can only be assayed using living cells (Harmsen, 2007).

Microbial biosensors are naturally occurring or engineered microorganisms producing a detectable signal upon environmental stimuli (Yagi, 2007; van der Meer and Belkin, 2010). Most of the sensors were designed based on bacteria in which a promoter induced by a molecule of interest drives expression of a reporter gene, producing a colorimetric, luminometric or fluorimetric output signal. In addition to the biotechnological workhorse *E. coli*, different soil‐borne bacteria such as *P. fluorescens*,* P. putida* or *S. aureus* have been engineered as whole‐cell biosensors to reduce the influence of native soil constituents (Renella and Giagnoni, 2016). To date, a variety of target analytes such as organic xenobiotics (naphthalene, BTEX [benzene, toluene, ethylbenzene and xylene], alkylsulphonates, polychlorinated biphenyls), heavy metals and metalloids (As, Cd, Zn, Ni, Cu, Cr, Cu), or nutrients and physiologically active molecules, can be detected by different kinds of whole‐cell biosensors (for in‐depth review, see (Yagi, 2007; van der Meer and Belkin, 2010), and references therein). Additionally, soil monitoring by whole‐cell biosensors that can detect molecules such as galactose, galactoside (Bringhurst *et al*., 2001) or nitrate (DeAngelis *et al*., 2005) also provide information about plant–microbe interaction and rhizosphere ecology for sustainable agriculture development. Genotoxins, chemical compounds causing harmful DNA damage, can be detected using the *umu*‐test, which is based on microbial DNA repair system (Biran *et al*., 2010).

For food monitoring, bacterial biosensors have been used to detect residual environmental pollutants, metabolizable products (e.g. ethanol, urea) and macro‐ and micronutrients (sugars, short‐chain fatty acids, amino acids, or vitamins). Bacterial biosensors also have been applied to the detection and identification of antibiotic residues to prevent allergies, toxicological effects and the emergence of antibiotic‐resistant bacteria. Engineered bacteriophages expressing a reporter gene upon infection are also a promising platform for identifying contamination in food or beverages by pathogenic bacteria and their toxins (Smartt and Ripp, 2010).

Beyond prokaryotic biosensors, yeast‐based biosensors, mostly using *Saccharomyces cerevisiae*, present several advantages including robustness, easy genetic manipulation and higher‐eukaryotic sensing modalities (Shimomura‐Shimizu and Karube, 2010). Yeast biosensors detecting environmental pollutants took advantage of yeast changes in respiration activity that can be monitored using a dissolved oxygen electrode that functions as an index of the level of degradable organic compounds present in the sample (Shimomura‐Shimizu and Karube, 2010; Jarque *et al*., 2016). These biosensors can detect biodegradable organics (Yudina *et al*., 2015), toxic heavy metals such as Cu^2+^ (Lehmann *et al*., 2000) or endocrine disrupting compounds (EDCs) (Schwartz‐Mittelman *et al*., 2005). The utilization of transcription factors activated by a molecule of interest and controlling a reporter gene are also a common strategy in yeast biosensor engineering (Shimomura‐Shimizu and Karube, 2010; Jarque *et al*., 2016). The similarity of DNA repair mechanisms between yeast and higher eukaryotes has also been used to expand yeast biosensors by detecting methylation‐based DNA damage (Moser *et al*., 2013) or general genotoxicity indicators (Benton *et al*., 2007).

## Whole‐cell biosensors for medical diagnostics and epidemiology

The gold standard method for detecting infectious agents remains culture isolation (Yager *et al*., 2008), which requires significant knowledge, training and time. Strategies for amplifying pathogens' nucleic acids are not adapted for low‐cost, point‐of‐care (POC) testing in low‐resource settings (Yager *et al*., 2008). Enzyme‐linked immunosorbent assay (ELISA)‐based tests are expensive and not well suited to use outside of state‐of‐the‐art laboratories (Fu *et al*., 2011). The majority of testing for infectious diseases in resource‐limited settings is thus performed by microscopy or agglutination tests.

For the development of POC *in vitro* diagnostics, bacteria able to sense quorum‐sensing molecules were engineered for detecting infections (Kumari *et al*., 2008). Whole‐cell biosensors have faced hurdles to operate in clinical samples due to unreliable operation and low signal‐to‐noise ratio in complex and heterogenous samples. In addition, WCBs usually have a limited signal processing capability precluding integration of several signals (e.g. multiple biomarkers) for accurate diagnosis. Recently, however, synthetic genetic circuits capable of signal amplification and multiplex processing have allowed the detection of pathological biomarkers in human clinical samples including abnormal glucosuria in the urine of diabetic patients (Courbet *et al*., 2015). Bacterial biosensors could further be extended to *in vivo* diagnosis (Slomovic *et al*., 2015). For example, engineered *E. coli* were tested in mouse models to indicate liver metastasis by producing detectable signals in urine (Danino *et al*., 2015), or to target cancer cells via cell‐surface expression of synthetic adhesins (Piñero‐Lambea *et al*., 2015). Another effective method is yeast‐based antibody display which has been applied to design highly sensitive POC technology for biosensing devices (Colby *et al*., 2004; Venkatesh *et al*., 2015; Aronoff‐Spencer *et al*., 2016). Antibody display was used to perform electrochemical detection of *Salmonella* or Hepatitis C virus (Aronoff‐Spencer *et al*., 2016). Another approach for yeast biosensor design uses conditionally stable ligand‐binding domains degraded in the absence of a cognate ligand to sense different small molecules (Feng *et al*., 2015). The use of the novo‐designed binders opens up the possibility of generating yeast biosensors for ligands with relevance in POC diagnostics.

## Cell‐free systems: going beyond living cells

While microbial biosensors hold a great deal of promise, they still have several limitations. For example, cell growth phase influences genetic circuit behaviour, adding noise to system and complicating reproducibility. Additionally, many compounds cannot cross the cellular membrane and are therefore not detectable using sensing machinery in the cytoplasm. Finally, many hazardous environmental contaminants remain toxic to the biosensor host organisms (Pellinen *et al*., 2004). One potential workaround is the use of cell‐free protein synthesis‐based biosensors. Cell‐free protein synthesis has long been used as a research tool and for recombinant protein expression (Nirenberg and Matthaei, 1961) and is now a common tool in synthetic biology (Perez *et al*., 2016). Briefly, cell‐free systems use the cellular machinery from harvested cell extracts, or individually purified compounds (Shimizu *et al*., 2005), to produce protein from DNA without the need for a living system. Unlike WCBs in which a large percentage of cellular resources is devoted to cell survival and replication, cell‐free systems allow all extant resources to be used in the biosensor. Cell‐free systems are more tolerant to a wide range of toxins and can detect mercury and antibiotics at higher concentrations than *in vivo* biosensors (Pellinen *et al*., 2004). Interestingly, recent work has shown that cell extracts containing these synthetic gene networks could be freeze‐dried on cellulose and stored more than a year at room temperature, while still remaining active when rehydrated. These paper‐based systems were used to engineer biosensors for Ebola and Zika viruses for POC (Pardee *et al*., 2014, 2016). CRISPR‐based systems were also used for highly sensitive detection of nucleic acid biomarkers (Gootenberg *et al*., 2017). Among the other advantages of paper‐based cell‐free system is the lack of a membrane, widening the range of detectable molecules.

## Challenges faced by microbially‐derived biosensors

**Figure 1:**
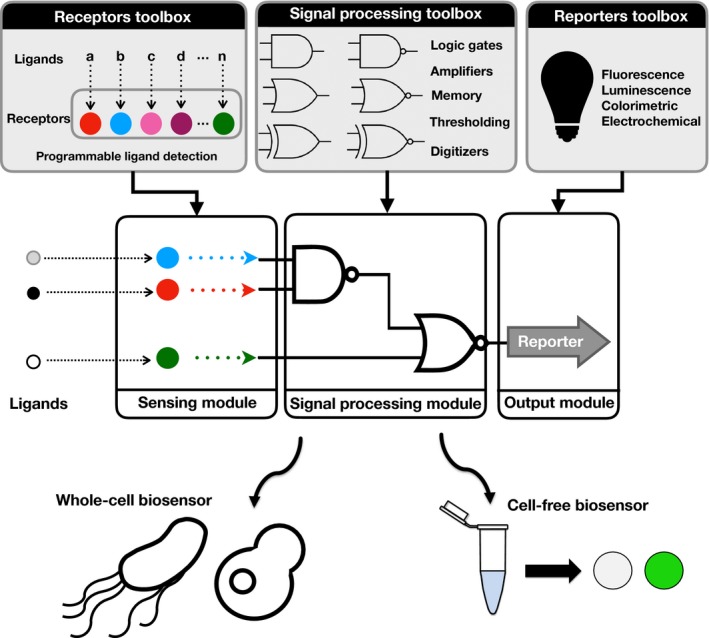
Next‐generation microbially derived biosensors. Synthetic biology research is providing an increasing number of biological parts enabling custom ligand detection, advanced signal processing and reporter output. These parts can be differentially composed into corresponding modules according to design specifications dictated by the envisioned application. Depending on the application constraints, the synthetic system obtained can be implemented either in a whole‐cell biosensor or in a cell‐free system operating on paper.

While microbial‐derived biosensors are useful tools for the detection of a wide range of analytes, these biosensors face several technical and societal challenges that have limited their widespread adoption and use. First, several technical limitations need to be solved. The natural promoters used in many biosensors can exhibit off‐target reactivity, responding not only to the molecule of interest but also to a group of compounds which interfere with promoter function (Cases and de Lorenzo, 2005). This is even more true for systems operating in complex, noisy samples like physiological fluids. In addition, the long response time for cell growth and reporter gene production complicates WCB usability for real‐time clinical monitoring (Yagi, 2007). Many applications, in particular clinical diagnosis, require multiplexed detection and processing of several biomarkers. Synthetic biology is providing tools that could address these problems (Fig. 1) (Courbet *et al*., 2016; Wei and Cheng, 2016). Also, using biosensors in low‐resource settings and harsh environments requires the development of convenient and adequate encapsulation formats, for example based on hydrogels (van der Meer and Belkin, 2010). Finally, a critical hurdle is that many ligands of interest cannot be detected because no receptor for them exists in nature. Therefore, current research should push towards developing synthetic receptors that could be easily tailored to detect many ligands of interest, ideally using versatile and well‐established antibody technologies.

Field release of microbial biosensors also faces several regulatory hurdles, resulting in long periods of waiting time before their use validation. Recently developed kill switches (Caliando and Voigt, 2015) and synthetic auxotrophies (Malyshev *et al*., 2014; Mandell *et al*., 2015) should support tighter control of microorganism spread. As an alternative, biosensors engineered using abiotic, cell‐free systems could help bypass these issues by providing a more controlled and non‐proliferating platform. Ethical issues are even more important for *in vivo* clinical applications, for which long and costly clinical assays and safety assessment need to be performed. Open and constructive debates need to be regularly conducted to define the societal and cultural context in which these technologies can be deployed (Webb *et al*., 2017).

## Conflict of interest

None declared.
